# Investigating the mechanisms underlying resistance to chemotherapy and to CRISPR-Cas9 in cancer cell lines

**DOI:** 10.1038/s41598-024-55138-x

**Published:** 2024-03-05

**Authors:** Francesca Tomasi, Matteo Pozzi, Mario Lauria

**Affiliations:** 1 CIBIO Department, University of Trento, Povo, Italy; 2 Fondazione Bruno Kessler, Povo, Italy; 3 Department of Mathematics, University of Trento, Povo, Italy; 4 Fondazione The Microsoft Research - University of Trento Centre for Computational and Systems Biology, Rovereto, Italy

**Keywords:** Cancer, Computational biology and bioinformatics, Drug discovery, Molecular biology

## Abstract

Cancer is one of the major causes of death worldwide and the development of multidrug resistance (MDR) in cancer cells is the principal cause of chemotherapy failure. To gain insights into the specific mechanisms of MDR in cancer cell lines, we developed a novel method for the combined analysis of recently published datasets on drug sensitivity and CRISPR loss-of-function screens for the same set of cancer cell lines. For our analysis, we first selected cell lines that consistently exhibit drug resistance across several classes of compounds. We then identified putative resistance genes for each class of compound and used inferred gene regulatory networks (GRNs) to study possible mechanisms underlying the development of MDR in the identified cancer cell lines. We show that the same method of analysis can also be used to identify cell lines that consistently exhibit resistance to the gene knockout effect of the CRISPR-Cas9 technique and to study the possible underlying mechanisms. In the GRN associated to the drug resistant cell lines, we identify genes previously associated with resistance (UHMK1, RALYL, MGST3, USP9X, and ESRG), genes for which an indirect association can be identified (SPINK13, LINC00664, MRPL38, and EMILIN3), and genes that are found to be overexpressed in non-resistant cancer cell lines (MRPL38, EMILIN3 and RALYL). In the GRNs associated to the CRISPR-Cas9 resistance mechanism, none of the identified genes has been previously reported in the admittedly sparse literature on the subject. However, some of these genes have a common role: APBB2, RUNX1T1, ZBTB7C, and ISX regulate transcription, while APBB2, BTG3, ZBTB7C, SZRD1 and LEF1 have a function in regulating proliferation, suggesting a role for these two pathways. While our results are specific for the lung cancer cell lines we selected for this work, our method of analysis can be applied to cell lines from other tissues and for which the required data is available.

## Introduction

With an estimated 10 million deaths worldwide in 2020, cancer is the leading cause of premature mortality and it lowers life expectancy in many countries^1,2^. Acquired resistance to anticancer treatments increase the morbidity and mortality of the malignant tumors^3^.

The ability of the cells exposed to a single drug to develop resistance is known as Multidrug Resistance (MDR)^4^. This is a major cause of chemotherapy failure^5^, and it is responsible for over 90% mortality of cancer patients^6^. There are several mechanisms that have been implicated in MDR^4^: enhanced efflux of drugs, genetic factors such as gene mutations, amplifications, and epigenetic alterations and/or deregulation of microRNAs^6,7^, growth factors, increased DNA repair capacity, and elevated metabolism of xenobiotics^6^. Additionally proposed mechanisms are alteration in target molecules^8^, deregulation of cell death mechanisms^7^, intratumor heterogeneity, cancer stem cell and enhanced plasticity^7^.

A number of efforts have focused on overcoming MDR by inhibiting drug transporters, however this approach has not been found to be sufficiently effective. Therefore it is urgent to develop novel systems to reverse drug resistance^9^. Novel approaches are employing the CRISPR-Cas9 system for different purposes, for example to edit MDR-related genes in a way that increases the sensitivity to anticancer treatment^10^, or in an exploratory fashion to identify potential targets for multi-drug treatments^11^. These novel approaches have to take into account the incompletely characterized CRISPR-Cas9 system limitations. As an example, functional resistance alleles occur when mutations prevent Cas9 cleavage at the intended cut site maintaining the function of the target gene. These alleles can pre-exist in the population or appear when the repair of the cleaved chromosome is mediated by NHEJ instead of HDR^12^.

The objective of this study was to gain insight into the mechanisms that confer multidrug resistance and those that confer resistance to CRISPR-Cas9 gene knockout to cancer cell lines. To achieve this goal, the analysis was divided into three distinct steps.

In the first step we identified cell lines exhibiting MDR by combining data on gene essentiality and drug sensitivity data Using such data, we selected gene-drug pairs that showed a statistically significant mutual relationship across a large number of cell types, presumably corresponding to pairings in which the gene is involved in the drug mode of action (for example as being the target of the compound). These relationships can be thought of as baseline models, each describing how the sensitivity to a certain drug depends on the varying levels of essentiality of the corresponding target gene observed across different cell lines. We then identified the cell lines that consistently departed from the baseline models by having the largest values of inhibitory drug concentrations, and we labeled these as the ones exhibiting MDR (henceforth called “resistant” cell lines).

In the second step, we studied the mechanisms that induce MDR in cancer cell lines by utilizing Gene Regulatory Networks (GRNs) that were inferred from the expression profiles and the genotype of the resistant cell lines. By restricting our network-based analysis to the genes that are differentially expressed in the resistantcell lines, the resulting GRNs provide valuable information about the relevant interactions between such genes and/or their encoded proteins^13^. The GRNs were constructed using FSSEM, a recently proposed method designed to simultaneously infer two related GRNs by integrating genetic perturbations and gene expression profiles obtained for two sets of samples to be compared^14^. In our study, the two sets of samples for which the GRN was inferred were represented by resistant and non-resistant cell lines.

Steps one and two were repeated with some minor modifications in order to study the mechanisms underlying the apparent attenuation of the CRISPR-Cas9 gene knockout effect that has been observed in some cancer cell lines. In this second iteration of the analysis, we focused on the contrast between CRISPR-Cas9 resistant and non-resistant cell lines.

In the last step, we identified the cell lines showing drug resistance using a different approach as a way of validating the results of the regression-based gene-drug pairing method.

The main contributions of this paper include the description of a novel method that can be used for identifying either drug-resistant or CRISPR-Cas9-resistant cancer cell lines, along with a network-based method of analysis of the underlying mechanisms. We have successfully identified relevant genes and suggested pathways associated with both forms of resistance. One of our incidental findings is that the mechanisms underlying drug resistance might exhibit tissue-specific characteristics.

## Results

### Integration of crispr and drug screening data

For our analysis we used two recently published datasets, one resulting from a genome-wide CRISPR-Cas9 loss-of-function screen and the other consisting of sensitivity measurements for anti-cancer compounds in cancer cell lines. The first dataset quantifies the essentiality of each gene (in terms of fold change depletion values) while the second reports drug lethality (in terms of IC50) for a large number of model cell lines. The CRISPR-Cas9 loss-of-function dataset contains dependencies profiles of 17,486 genes across 908 different cell lines and is publicly available from the DepMap portal^15^. Experimental data on drug sensitivity screens was obtained from the Genomics of Drug Sensitivity in Cancer (GDSC) project, which includes data obtained following two slightly different protocols (datasets GDSC1 and GDSC2). A matrix including the IC50 values for 565 compounds across 988 cell lines was created by merging and reshaping data for both experimental protocols. We extracted the cell lines common to both datasets, obtaining a subsample comprising 542 cell lines.

Our approach to the integration of these two types of data is inspired by the work of Gonçalves et al.^16^ in which the authors sought to identify significant relationships between the effects of gene knockout and the administration of a drug that targets the same gene or the corresponding protein. Our method diverges from that of Gonçalves et al. as we pursue a different objective. In our approach, the initial discovery of significant gene/drug relationships serves as an intermediate step preceding additional in-depth gene level analysis.

**Figure 1:**
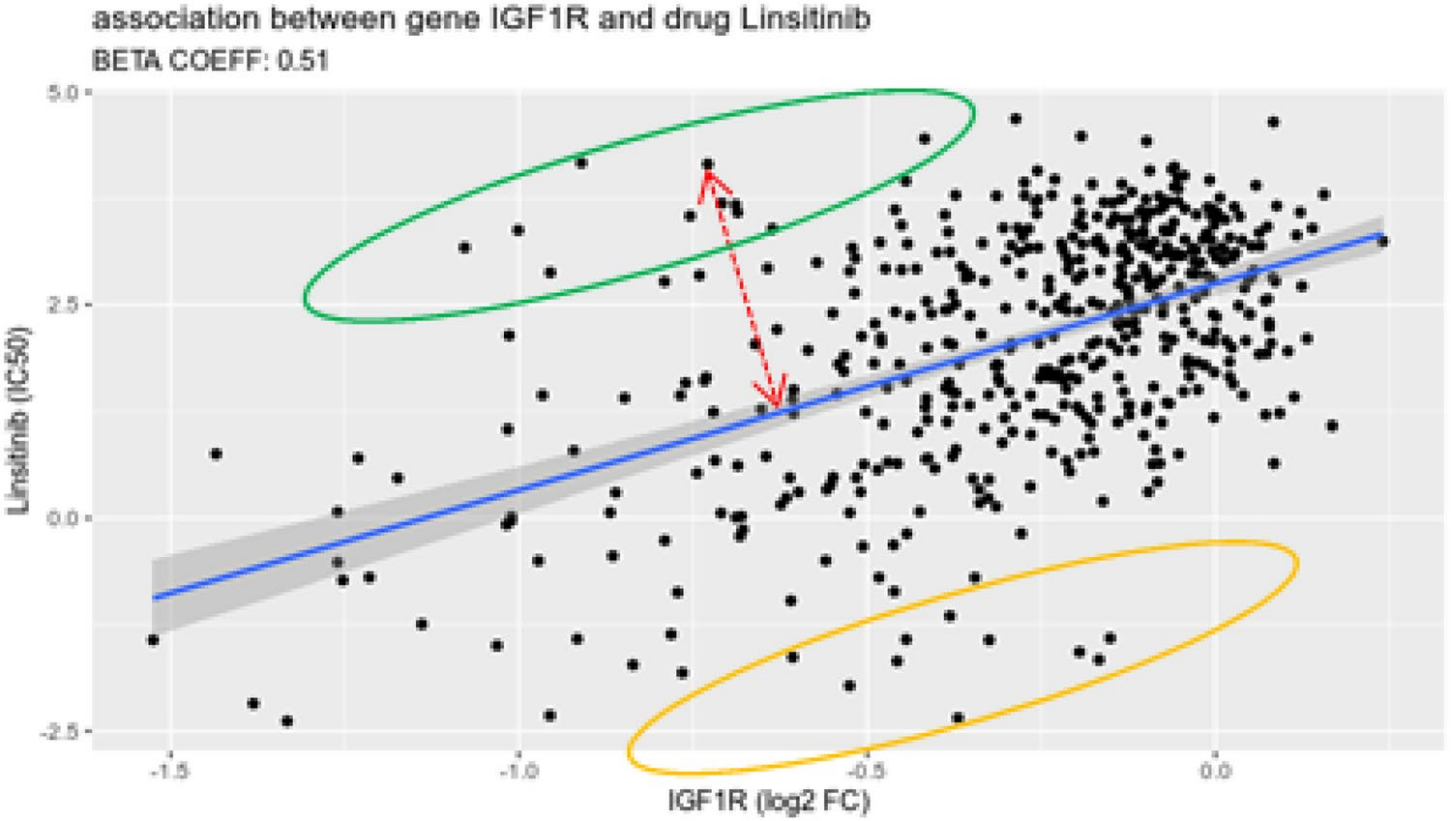
Scatter plot of the gene-drug association for the IGF1R gene and the Linsitinib drug. Each dot represents a cell line; the *blue* line is the regression line. For each cell line we consider its distance from the line (*red* dashed line), and we are particularly interested in those consistently showing a large distance across associations. We conduct separate analyses for the cell lines positioned above (referred to as ‘positive distance’) and below (referred to as ‘negative distance’) the regression line. These two sets correspond to the cell lines we classify as drug-resistant (represented by the *green* oval) and CRISPR-Cas9 resistant (represented by the *orange* oval), respectively.

In simple terms, the starting point of our analysis was the identification of gene-drug pairs showing a statistically significant mutual relationship across a large number of cell types. For each gene-drug pair, a scatter plot was created with the *x* axis representing the log of the fold change from the CRISPR-Cas9 gene knockout and the on the *y* axis representing the IC50 value for the drug; in this plot each cell line is represented by a point whose coordinates are the fold change and IC50 values for the line according to the two datasets (Fig. 1). We applied regression analysis to each plot to verify the existence of a meaningful linear relationship and selected those whose statistics achieved a satisfactory level of significance (adjusted *p*-value < 0.1). As expected, only a subset of gene-drug combinations showed a significant relationship, presumably those involving a gene that is the target of the drug or a closely related one.

**Figure 2:**
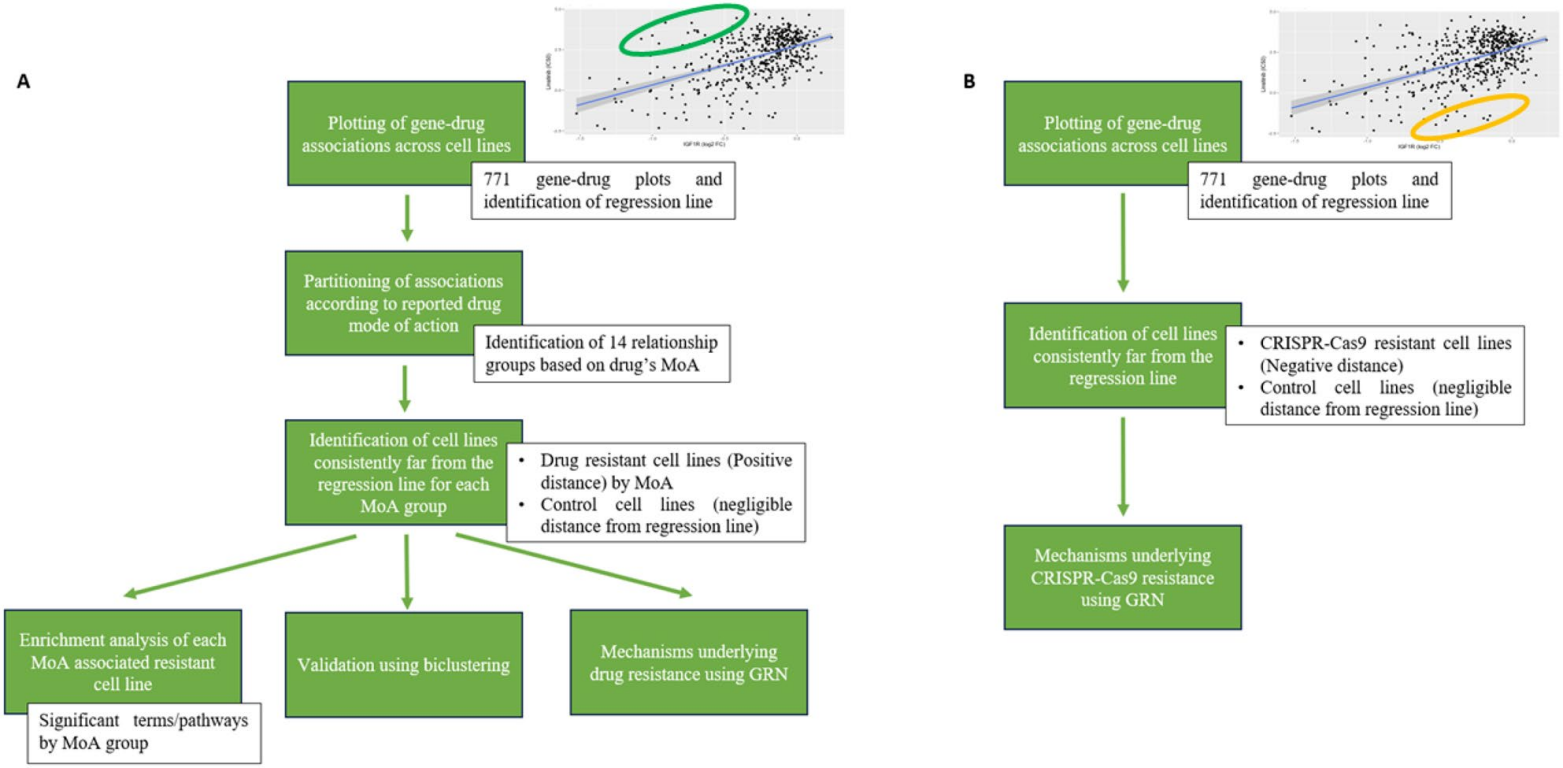
(**A**) Scheme describing the main steps of the analysis of drug resistance mechanisms. (**B**) Scheme representing the main steps of the analysis of CRISPR-Cas9 resistance mechanisms.

Next, for each cell line we recorded its distance from the line, and we identified those consistently showing a large distance across the significant gene-drug pairs. We conducted separate analyses for the cell lines positioned above (referred to as ‘positive distance’) and below (referred to as ‘negative distance’) the regression line (Fig. 1). These two sets correspond to the cell lines we classify as drug-resistant and CRISPR-Cas9 resistant, respectively. The steps of our analysis for the two sets of cell lines are summarized in Fig. 2.

In greater detail, the relationships between CRISPR loss-of-function score and drug sensitivity measurements were modelled using linear mixed-effect models (LMM) following the procedure described in Gonçalves et al.^16^. Briefly, a linear model was fitted to each drug/gene pair using the vector of drug response IC50 values across cell lines as the response variable with the following variables as covariates: (i) binary variables indicating the institute of origin of the cell line CRISPR-Cas9 screen; (ii) principal component 1 of the drug response data set which is a correlative of cell lines growth rate; and (iii) growing conditions (adherent, suspension or semi-adherent) represented as binary variables. The random effects are represented by the CRISPR-Cas9 score similarity matrix of the samples. All pairwise associations between 426 compounds and 17,486 genes were tested, resulting in a total of 7,449,036 associations. The statistical significance of each association was assessed by performing likelihood-ratio tests between the full model and an alternative model which omits the CRISPR gene fitness scores among the covariates. P-value adjustments for multiple testing was performed by using the Benjamini–Hochberg false discovery rate (FDR). 771 significant association between drug response and gene fitness scores were obtained by considering an FDR-adjusted *p*-value < 10%.

The next step considers a graphical representation of each linear model in which each point corresponds to one of the 542 cell lines and the regression line from the model is used to separate the plane in two regions, one above and the other below the line. Cell lines whose distance from the regression line approaches zero exhibit a proportional response to the treatment when the gene is knocked out. Cell lines with a positive distance from the regression line (that is, falling in the region above the line) are responsive to the gene knockout, meaning that the gene has a certain importance for the survival of such cells, while requiring relatively high drug concentrations to affect the same gene. The existence of a mechanism that impairs the drug's mode of action inside the cell line may be the source of such relatively low sensitivity. Cell lines with negative distances (that is, falling in the region below the regression line for that gene/drug pair) have a good response to pharmaceutical therapy but exhibit a relatively high value of CRISPR induced fold change, suggesting an impairment of the CRISPR-Cas9 action. To quantify the discordance from the expected response of each cell line, the distance of each point from the regression line was computed for each cell line for all the 771 significant associations, resulting in a 771 × 542 distance matrix.

**Figure 3:**
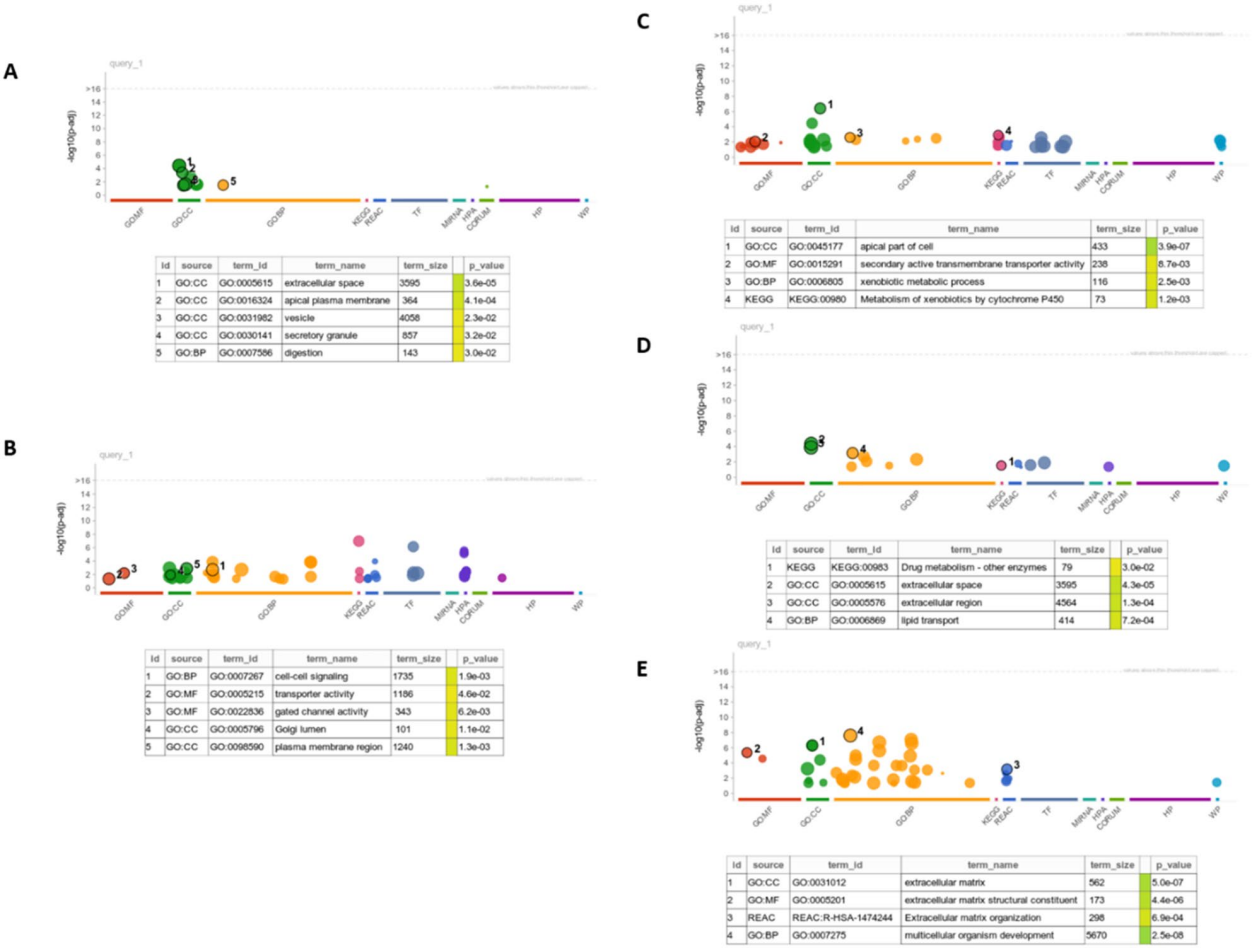
Summary of the most significant terms from the enrichment analysis of upregulated genes resulting from the clustering of gene-drug associations according to the pathway targeted by the drugs. (**A**) DNA regulation pathway. (**B**) Genome integrity pathway. (**C**) Mitosis pathway. (**D)** PI3K pathway. (**E**) ERK MAPK pathway.

Next step was to find those cell lines that exhibit a lowered sensitivity to drug response relative to gene knockout sensitivity (positive distance) for a large number of associations. To identify such cell lines we computed their median distance across associations and studied the ones with the larger median values. Two groups of cell lines were created: 1) 10% of cells with the highest median distance (drug resistant cell lines), and 2) 10% of cell lines with distance closest to zero (control cell lines). A differential gene expression analysis was performed and the resulting differentially expressed genes were short-listed based on an adjusted *p*-value < 0.05 and an absolute log_2_(FoldChange) > 3. When a functional enrichment analysis on the resulting genes was performed, no significant results were found. We reasoned that considering the whole set of associations in computing the median would produce satisfactory results only if the same genes were responsible for the resistance mechanisms in all of them. Therefore, in order to correctly study mechanisms conferring resistance, a better approach would be to partition the significant gene/drug associations according to the drug mode of action. Based on drug information available in our dataset, a set of putative target pathways (PI3K-mTOR, apoptosis, DNA replication, genome integrity, RTK, ERK-MAPK, cell cycle, mitosis, other kinases) was considered to identify DEGs in a pathway-specific manner, followed by a corresponding functional enrichment analysis. Following this approach, statistically significant results were obtained from the functional analysis, with enriched GO terms related to drug and multidrug resistant mechanisms. Some representative findings are reported for the following pathways: DNA regulation, genome integrity, mitosis, PI3K, ERK-MAPK (Fig. 3A–E). Among them, terms related to putative mechanisms reconducible to drug resistance can be recognized. In particular, the upregulation of drug metabolism as well as mis-regulations of vesicles, transporters and extracellular matrix are mechanisms which are well known to be related to multidrug resistance.

### Resistance to drug treatment and resistance to CRISPR-Cas9

To gain some insight on the specific mechanisms involved in drug and in CRISPR resistance in cancer cell lines, we proceeded to study the differences between resistant and non-resistant cell lines separately for each of the two types of resistance. We first identified genes whose expression differ between the resistant and non-resistant cell lines, then combined this list with the respective SNP profiles to infer GRNs simultaneously for the two groups using the FSSEM network inference tool. Using a regression-based approach, FSSEM aims at identifying regulatory interaction between genes by comparing their expression levels. The role of the SNPs in altering the expression levels of the associated genes is taken into account to infer the causality of the relationship (i.e. the direction of the regulation). By inferring the interaction networks simultaneously for the resistant and non-resistant groups, this approach increases the sensitivity to the differences between the two GRNs. MatrixEQTL is the tool used to select the subset of SNP-gene pairs that show a significant association based on the cell line data.

#### Investigating genes involved in the resistance to drug therapy

We divide the cell lines in resistant and non-resistant to chemotherapeutics and according to the tissue of origin (Supplementary Table 2). To remove the confounding effect of the heterogeneity of the tissues of origin of the cells (effect clearly visible in Supplementary Fig. 1), we restricted our analysis to lines derived from the same tissue. The tissue associated to the highest number of cell lines (Lung) was selected. For the same reason, only cell lines coming from a primary tumor were considered, with the objective of excluding all possible mutations that the cell lines might acquire in the process of becoming metastatic.

**Figure 4:**
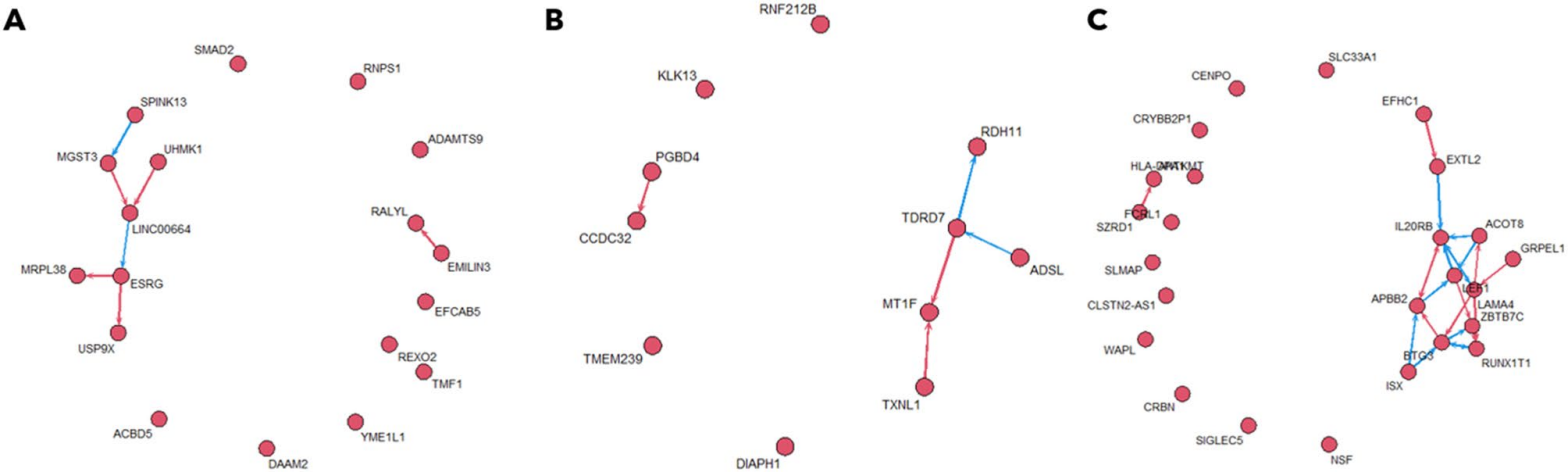
Gene regulatory networks (GRNs) highlighting differences between two cell groups. Up-regulated edges are colored in red and down-regulated edges are colored in blue. Disconnected genes are those for which there are no differences in regulatory interactions between the two groups of cells. (**A**) Difference GRN obtained using drug resistant vs drug non-resistant Lung cell lines. (**B**) Difference GRN obtained using drug resistant versus drug non-resistant Large Intestine cell lines. (**C**) Difference GRN obtained using CRISPR resistant versus CRISPR non-resistant Large Intestine cell lines.

After the analysis of the DEGs, we ended up with 1615 genes. After an additional filtering step in which we identified the cis eQTL and their associated genes, we ended up with 18 genes and 22 SNPs which are used to build the GRN (Fig. 4A).

The genes connected by an edge in the GRN were studied individually (Supplementary Table 4). Five genes (UHMK1, RALYL, MGST3, USP9X, ESRG) are directly related with the drug resistance phenotype, while the remaining four genes (SPINK13, LINC00664, MRPL38, EMILIN3) do not have a known direct relationship with drug resistance in cancer, but they belong to families of genes that are known to be involved in drug resistance.

Investigating the expression of the genes, we found out that 6 are overexpressed in resistant cell lines, while EMILIN3, MRPL38, and RALYL are overexpressed in the non-resistant cell lines. It is possible that EMILIN3 and MRPL38 are not directly involved in the development of drug resistance in cancer; the fact that we were unable to find any information in the literature linking them to drug resistance reinforces this hypothesis. RALYL instead is known to increase resistance to cisplatin in HCC, and we surmise that this mechanism is not used in Lung cancer.

After studying each gene of the subnetwork, the connections (Supplementary Table 5) were studied in turn by uploading the two genes on STRING and identifying possible intermediate genes (i.e. the additional intervening genes needed to establish a path between them). For the first edge EMILIN3 → RALYL two intermediate genes (ZNRD1, BARX2) were found on STRING.The role of these two genes is similar, as both are involved in identical protein binding activity. For the second connection SPINL13 → MGST3, three intermediate genes (SPINK9, SOD3, PTGES) were found on STRING.

The other interactions were between a non-coding gene and a coding gene therefore the edges could not be studied on STRING. Searching the literature we found that UHMK1 and MGST3 are both connected to LINC00664 and have a top transcription factor binding site in common: Pbx1a. Additionally, they are on the same chromosome (Chr 1). We also found that MRPL38 and USP9X, both connected to ESRG, have a top transcription factor binding sites in common: Egr-4. On the basis of these results and knowing that lncRNAs have been shown to assume regulatory roles by acting as co-factors to modify the activity of transcriptional factors^17–19^, we hypothesize that lncRNAs can interfere with the expression of the genes through the transcription factor binding site. This could be valid for the ESRG gene, given thatthe direction of the regulation goes from ESRG to MRPL38 and USP9X, and for the LINC00664 → ESRG regulatory interaction. However for the edges connecting MGST3 and UHMK1 to LINC00664, this is not possible and the interaction must have a different nature.

It is known that UHMK1 induces resistance by interacting with STAT3. This protein is a signal transducers and activators of transcription.^20^. STAT3 could interact with the promoter through the binding site of a lncRNA (HOXD-AS1) and in this way regulating it^21^. To explain the edge between UHMK1 and LINC00664, it is possible to assume that STAT3 can regulate also the expression of LINC00664. This hypothesis is not supported by known information and should be validated in the future. For the interaction between MGST3 and LINC00664, instead, no information in literature was found.

The analysis performed on Lung tumor cell lines was repeated using cell lines derived from the Large Intestine.

In this case we ended up with 1250 DEGs, and after the filtering steps for the cis eQTL identification we obtained 11 genes and 11 SNPs which were then used to build the GRN (Fig. 4B) with the FSSEM algorithm.

None of the genes identified using Intestine primary tumor cell lines are present in the GRN previously obtained using Lung primary tumor cancer cell lines. If confirmed this result would suggest that different mechanisms causing resistance to drug therapy are at work in the two tissues.

#### Investigating genes involved in the resistance to CRISPR-Cas9

Following the same steps outlined before, the cell lines were divided in resistant and sensitive to CRISPR-Cas9 and according to the tissue of origin. Only the cell lines from Lung and the ones coming from a primary tumor were selected for subsequent analysis.

We identified 1329 DEGs and after running *MatrixEQTL* we obtained 25 genes and 31 eQTLs which were used to build the GRN (Fig. 4C) with the FSSEM algorithm.

The GRN obtained consists of 25 genes. Among these, 14 of them (ACOT8, BTG3, EFHC1, ZBTB7C, EXTL2, SZRD1, APBB2, LAMA4, LEF1, IL20RB, ANTKMT, GRPEL1, RUNX1T1, ISX) are connected in a subnetwork and, as for the other GRN, they were studied individually (Supplementary Table 6). Of these 14 genes, some information about CRISPR-Cas9 related studies was found for 9 of them.

Regarding the role of the GRN genes, we noted that there are some that share the same function: APBB2, RUNX1T1, ZBTB7C, and ISX have a role in regulating transcription, while APBB2, BTG3, ZBTB7C, SZRD1 and LEF1 have a role in regulating proliferation. We hypothesize that these pathways are involved in the development of the inability of the CRISPR-Cas9 mechanism of action.

After studying individually each gene of the subnetwork, the connections between genes were studied in turn (Supplementary Table 7). As done before, the two genes connected by an edge in the GRN were uploaded on STRING. The STRING connections are undirected while the edges in the GRN obtained by FSSEM are directed, however in our analysis we neglected the direction of the edge and report any known relationship between the genes regardless of their causality relationship. In 10 cases one additional intermediate gene is needed to connect the starting gene to the end gene, while in 13 cases two intermediate genes are needed.

### Overlap between tissue-specific DEGs and DEGs divided by MoA

As described before, pathways involved in drug mode of action were identified and associated with cell lines based on the drug for which the lines exhibited resistance. DEGs were identified for each of the pathway-specific cell line groups. Using a hypergeometric test, we wanted to check if there is a significant overlap with the DEGs identified for the construction of the GRNs.

The genes identified for each MoA are compared with the DEGs obtained from the Lung analysis and from the Large Intestine one. The results of the hypergeometric test are summarized in Tables 1 and 2.

**Table 1:** Overlap using DEG obtained between lung—primary tumor resistant cancer cell lines and lung—primary tumor non-resistant cancer cell lines.

MoA	Length list	Overlap	*P*-value
Apoptosis	17	1	0.5292458
Cell cycle	105	7	0.1710078
DNA regulation	68	3	0.5698679
ERK-MAPK	234	11	0.4351326
Genome integrity	391	17	0.5296166
**Mitosis**	290	25	**0.0009258557**
Other kinases	131	9	0.12022
PI3K	103	5	0.4631532
RTK	141	5	0.7360873

Significant values are in [bold].

**Table 2:** Overlap using DEG obtained between intestine—primary tumor resistant cancer cell lines and intestine—primary tumor non-resistant cancer cell lines.

MoA	Length list	Overlap	P-value
Apoptosis	17	0	1
**Cell cycle**	105	7	**0.06344614**
**DNA regulation**	68	5	**0.07798273**
ERK-MAPK	234	5	0.8961865
Genome integrity	391	14	0.440028
Mitosis	290	6	0.926139
Other kinases	131	4	0.6500757
PI3K	103	1	0.9703843
RTK	141	5	0.5134716

Nearly significant values are in [bold].

Table 1 shows that the MoA involved in the resistance in the Lung is the mitosis with a p-value = 0.0009258557; Table 2 shows that for the Intestine there are no MoAs that achieve significance with respect to a p-value threshold of 0,05, however the cell cycle and the DNA regulation are just above threshold. We also note that the GRN built using the Lung cancer cell lines and the one built using the Intestine cancer cell lines do not have genes in common. A possible explanation is that different mechanisms are responsible for drug resistance in different tissues. However this observation would need to be confirmed in a study with a larger number of cell lines.

The 25 genes that compare in the overlap between DEGs of Lung and mitosis are TBX21, CXCL5, IGFBP1, KIF1A, NPSR1, CLPSL1, SPINK1, PROK2, NCKAP1L, GAL3ST1, LRRN3, PDZD3, BEND4, TM4SF20, RXFP1, CCDC144NL, ATCAY, RPRML, ACTN2, PTPRC, MMP3, PGR, AEBP1, SNAP91, NPY. The 7 genes from the overlap between the DEGs of Intestine and Cell Cycle are RNASE1, MUCL1, LY6G6D, ATP1A4, CSF3R, SLC7A4, ST6GAL2, while the 5 genes from the overlap between the DEGs of Intestine and the DNA regulation are GLI1, TMEM72, ITGAD, DMBT1, DNAH9.

These results are consistent with our hypothesis that different tissues use different MoA to cause resistance to chemotherapeutics.

### Validation using biclustering

The target of the next step was to validate the results obtained with the distance from the regression line method using a completely different approach. We used biclustering to identify cell lines in which the Multidrug Resistance (MDR) genes are enriched and therefore classify them as drug resistant cell lines. The biclustering is done with the three datasets described in *Materials and Methods* and the results are summarized in Supplementary Table 1. The subsequent analysis was restricted to data in the form of TPM values downloaded from Cell Model Passport, which was normalized as log_2_(TPM+1), since this represents the combination of dataset plus normalization that produces the best significance values according to the statistical test employed.

**Figure 5:**
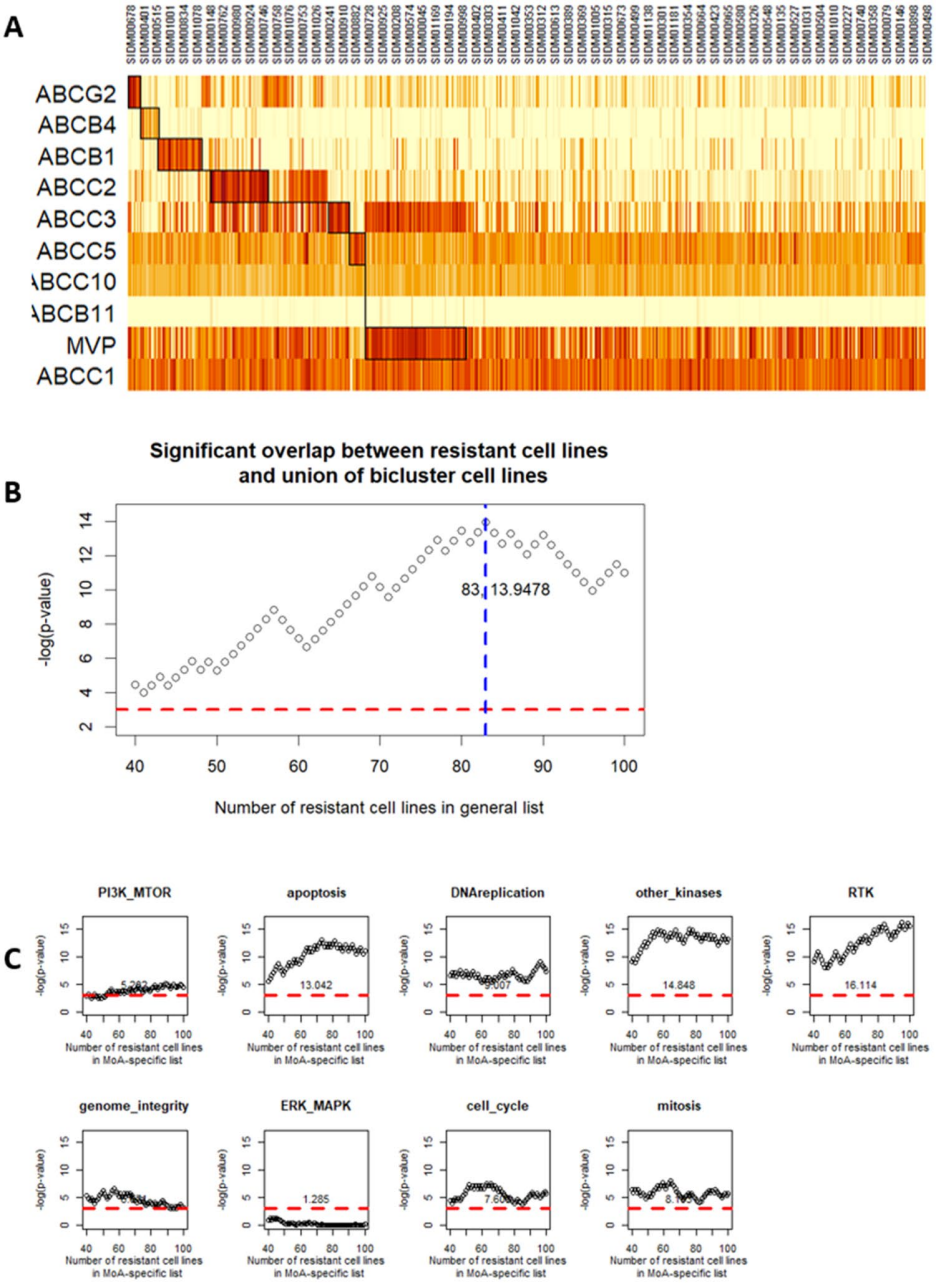
Biclustering and Chi-square test results obtained using data downloaded from Cell Model Passport with normalization log_2_(TPM + 1) (**A**) Biclustering using the subset of Multidrug resistance (MDR) genes and the ISA biclustering method. (**B**). (**C**) The *red* line corresponds to − log(0.05) = 2.995732. Scores above that line are significant. The vertical *blue* line corresponds to the max of the − log(*p*-value). Next to the blue line are the numbers representing how many top N cell lines are needed to reach the max − log(*p*-value), and the max value itself. (**B**) Chi-square test results. (**C**) Chi-square test performed on the resistant cell lines identified for each mechanism of action (MoA) vs the sum of all resistant cell lines identified with the ISA biclustering method.

In Fig. 5A (and Supplementary Fig. 2) it is possible to visualize some of the clusters obtained. The cell lines grouped inside the clusters are the ones identified as drug resistant and used to perform the Chi-square test. This test was used to study the overlap between the cell lines obtained with the biclustering and the cell lines obtained according to the distance from the regression line method. The Chi-square test is performed multiple times with different combination of lists of resistant cell lines.

#### Comparison #1

Firstly, Chi-square test is computed using the union of all cell lines that appear in at least one ISA cluster and the resistant cell lines previously identified. Here we consider a variable number of resistant cell lines between top 40 and top 100 with a positive distance from the regression line and perform Chi-square test for all cases. Figure 5B (and Supplementary Fig. 3) shows how the p-value obtained from the Chi-square test changes according to the number of drug resistant cell lines. In this way we were able to identify the maximum -log(p-value) = 13.9478 that corresponds to the top N = 83 cell lines identified as resistant.

#### Comparison #2

Lastly, Chi-square test was computed using the union of all cell lines that appear in at least one ISA cluster and the list of resistant cell lines for a specific mechanism of action (MoA) (Fig. 5C and Supplementary Fig. 4). It can be seen that the -log(p-value) for all MoAs, with the only exception of the ERK-MAPK pathway, are above the threshold. All MoAs previously identified appear to work well.

## Discussion

To gain insights into the specific mechanisms of MDR in cancer cell lines, we developed a novel method for the combined analysis of anti-cancer drug sensitivity measurements and genome-wide CRISPR loss-of-function screens performed on the same set of cancer cell lines. While the data used in our study was previously employed to investigate drug mechanism-of-action across a large collection of drugs and of cell lines, in the first part of our analysis we used it to identify a subset of cell lines consistently exhibiting drug resistance. Crucially, we expanded our analysis by considering all significant gene-drug interactions and not only the ones between a drug and its putative target gene. In addition, our study considers that the difference between sensitivity to drug treatment and sensitivity to gene knockout could also be due to an impairment of the CRISPR-Cas9 mechanism of action. The starting point of our analysis is the systematic modeling of the association between gene essentiality (sensitivity of the cell to the loss of function of the gene) versus drug sensitivity for a large number of cell lines. In each association a cell line is represented by two coordinates: (1) the wild-type vs CRISPR depletion fold change resulting from the knockout of the gene in that cell line, and (2) the IC50 resulting from the administration of the drug to the same cell line. By modelling the associations as a linear relationship, specifically as a LMM-type regression, we identify cell lines that deviate from the predicted response, given the observed fold change.

Computing the distance of each cell line from the regression line in each of the 771 significant associations enabled us to (1) identify cell lines which consistently exhibit positive distance, and (2) analyze these cell lines to extract MDR features and genes.

In order to elucidate MDR mechanisms we first clustered gene/drug associations by drugs’ pathways of action, the rationale being that in general the resistance mechanism should be presumed to be pathway specific. We then computed the median value for each cell line separately for each cluster. The resulting list of median distances was sorted and cell lines with top 10% positive median distance and 10% of cell lines with smallest median distance were selected for subsequent differential gene expression. The functional enrichment analysis of the DEGs yielded biologically relevant and statistically significant results. Multidrug resistance mechanisms were identified, such as an upregulation of the drug metabolism and transporter activity.

We then investigated the mechanisms that induce the resistance to drug treatment and to CRISPR-Cas9 in cancer cell lines. This was done building GRNs that are specific for the group of cell lines under study. We inferred the GRNs using the FSSEM tool, an innovative method designed to combine the information from expression and genotypic profiles to obtain networks that are hopefully highly descriptive of the gene regulation specificities of the lines under study. For our network-based analysis we took advantage of two innovations implemented in this tool: (1) the combined use of two different profiles (genotype and transcriptome) for the same cell lines, and (2) the comparative analysis of two groups of cells, namely resistant vs non-resistant, to highlight their differences. Ideally these two innovations converge toward the identification of regulatory interactions that are very relevant for the types of resistance studied, with the exclusion of confounding effects such as those represented by tissue type and disease status.

The first GRN is identified in connection with drug resistance. UHMK1, RALYL, MGST3, USP9X, ESRG are directly related with the drug resistance phenotype, while SPINK13, LINC00664, MRPL38, EMILIN3 do not have a direct relationship with drug resistance in cancer, but there are other genes that belong to the same family that are known to be involved in drug resistance. MRPL38, EMILIN3 and RALYL are overexpressed in non-resistant cancer cell lines. We suppose these three genes are not simply involved in the development of the resistance.

In addition to the role in the development of drug resistance of single genes, the edges representing their interaction are studied. To connect RALYL to EMILIN3, two intermediate genes are found in STRING, while to connect SPINL13 to MGST3, three intermediate genes are found. All the other connections, instead, involve two lncRNAs: ESRG and LINC00664. Based on previous information about lncRNAs, it is possible that lncRNAs regulates the expression of the other genes connected in the subnetwork. This could be valid for the ESRG gene since the direction of the inferred regulation is from ESRG to MRPL38 and USP9X and for the edge LINC00664 → ESRG. Instead, the interaction described by the edges that go from MGST3 and UHMK1 to LINC00664 should have a different nature. In literature there is no information about an interaction between LINC00664 and MGST3 and between LINC00664 and UHMK1. It is possible that these links are still not sufficiently studied, but some hypothesis could be suggested. It is known that UHMK1 induces resistance by interacting with STAT3 which is a signal transducers and activators of transcription^20^ and it could interact with the promoter through the binding site of a lncRNA and regulates it^21^. It is conceivable that STAT3 can also regulate the expression of LINC00664. This hypothesis is not supported by literature and should be validated. For the interaction between MGST3 and LINC00664, instead, no information was found.

Most of the edges of the GRN involve a lncRNA (LINC00664 or ESRG). It is already known that lncRNAs can cause resistance to anticancer therapy^18^. This suggests that lncRNAs have a role in the development of the resistance together with cancer stem cells. Cancer stem cells are known to cause resistance to chemotherapeutics^22^, and the presence of the gene ESRG in the GRN underscores the presence of these cells in the cancer population. However, the specific role of LINC00664 in drug resistance is not yet known and it is left to future investigations.

The same analysis was repeated using the cell lines from the Large Intestine. The newly obtained GRN does not have genes in common with the GRN obtained using Lung cancer cell lines. This result suggests that different mechanisms are responsible for resistance to chemotherapeutics in different tissues.

To study CRISPR-Cas9 resistance we built a dedicated GRN. Some information about CRISPR-Cas9 related studies was found for 9 genes, while for the remaining 5 no connections with CRISPR-Cas9 system was found in literature. For none of them a direct explanatory relationship between the gene and CRISPR-Cas9 resistance could be identified.

Studying the role of the genes present in the differential GRN, it is possible to note that there are genes that share the same function: 4 genes have a role in regulating the transcription and 5 genes have a role in regulating proliferation. We can assume that these two pathways have a role in the development of CRISPR-Cas9 resistance.

Next the edges were examined by searching for known interactions. The two genes connected by each edge were uploaded on STRING. In 10 cases one additional intermediate gene was needed to connect the starting gene to the end gene, while in 13 cases two intermediate genes were needed.

In the last part, we sought to validate the results obtained with the method previously described using a completely different strategy. Using a biclustering algorithm, cell lines for which the expression of some of the known MDR genes is above average were clustered together. The cell lines present in the clusters were labelled as drug resistant according to the new method. Next we verified that there was a statistically significant amount of overlap between the two sets of drug resistant cell lines identified with the two methods. The existence of a significant concordance for all MoA previously identified gave us confidence in the validity of the identification of resistant cell lines using the aggregated distance from the regression line method.

One critical detail of this procedure is how the list of MDR genes used for the biclustering is created. Such list was assembled from literature, collecting the genes already implicated in the development of resistance in cells. The genes identified in this way were those involved with Drug Efflux, one of the known mechanisms causing MDR. It is however possible that in some of the cancer cell lines the resistance is due to still uncharacterized mechanisms and would go undetected by this approach. Therefore we employed the biclustering as a validation method, and we relied on the distance-based method for discovery.

Another crucial detail regards the number of cell lines to be included in the resistant group in the first step of the distance-based method. In designating some of the cell lines as drug resistant, we used an arbitrarily chosen 10% value as the percentage of top cell lines by positive median distance to be included in the list. Based on the analysis that we performed using a variable number of top cell lines, we have some indication that including a higher number of cell lines might give better results.

## Conclusions

Multidrug resistance (MDR) stands as a primary factor contributing to the ineffectiveness of chemotherapy regimens. Gaining insights into the intricate mechanisms responsible for MDR in cancer holds significant promise for the discovery of novel anticancer therapies. The utilization of the CRISPR-Cas9 technique not only facilitates the identification of genes implicated in MDR mechanisms but also offers a potential means to overcome drug resistance challenges. Identifying mechanisms and genes which can impair the mechanism of action of CRISPR will be instrumental in enhancing the performance and accuracy of large-scale screenings.

The initial phase of this study introduces a novel methodology that is readily adaptable for the identification of either drug-resistant cancer cell lines or CRISPR-Cas9-resistant cell lines. Differential gene expression analysis of these interesting cell lines unveils terms significantly associated with either resistance mechanism in cancer.

To help elucidate drug and CRISPR-Cas9 resistance mechanisms, we employ the FSSEM method to construct Gene Regulatory Networks (GRNs). In the context of drug resistance, five genes (UHMK1, RALYL, MGST3, USP9X, ESRG) in the GRN exhibit direct relationship with drug resistance, while the remaining genes (SPINK13, LINC00664, MRPL38, EMILIN3 are linked to the resistant phenotype through their membership in gene families known to be associated with drug resistance.

In the CRISPR-Cas9 network analysis, none of the identified genes exhibit an association with CRISPR-Cas9 resistance. Some genes share common cellular functions, such as APBB2, RUNX1T1, ZBTB7C, and ISX, which regulate transcription, and APBB2, BTG3, ZBTB7C, SZRD1 and LEF, implicated in proliferation regulation. These pathways may potentially influence the efficacy of the CRISPR-Cas9 mechanism.

The edges within the GRNs highlight regulatory interactions relevant to drug resistance, encompassing both direct and indirect interactions mediated by other genes. While the roles of intermediate and non-coding genes remain unexplored in this study, their potential significance warrants future investigations. Notably, certain long non-coding RNAs (lncRNAs) such as ESRG and LINC00664 are identified in the Lung drug-resistant GRN. ESRG appears to regulate the two genes to which it is connected through their transcription factors, while the connections of LINC00664 require further exploration.

Finally, the lack of overlap between the GRNs derived for Lung and Intestine resistance underscores the tissue-specific nature of chemotherapeutic resistance mechanisms. Consequently, future research efforts should be designed by taking into account this tissue-specificity. It's important to note that this result should be further validated; a limitation of this study lies in the relatively small number of cell lines of homogeneous tissue origin for which data is available. Expanding the dataset could augment the analytical power, potentially yielding more extensive and comprehensive GRNs.

## Materials and methods

### Integration of CRISPR and drug screening data for investigating drug mode of action and multi-drug resistance

#### CRISPR data

CRISPR data are those described in the Gonçalves et al. study and are publicly available from DepMap portal^23^. This data contains dependency profiles of 17,486 genes across 908 different cell lines which spans 26 tissues and 42 different cancer types and can be downloaded from https://cog.sanger.ac.uk/cmp/download/integrated_Sanger_Broad_essentiality_matrices_20200402.zip.

Of the files contained in this dataset, we have used *CRISPRcleanR_FC.txt* to perform our analysis.

The gene level fold changes were quantile normalised per sample and then median scaled using known lists of essential and non-essential genes such that essential genes have a median log_2_(FoldChange) = − 1 while non-essential genes have a median log_2_(FoldChange) = 0.

#### Drug sensitivity data

Experimental data on drug sensitivity screens can be found in the Genomics of Drug Sensitivity in Cancer (GDSC) project^24^. This database is part of an initiative from a joint effort between the Sanger and Broad institute, called the Cancer Dependency Map^25^. The GDSC dataset is composed of two main types of data: GDSC1 and GDSC2^26,27^ which differ by screening procedures and some of the drugs screened. The specific versions of the data we used can be found at the following URLs: https://ftp.sanger.ac.uk/project/cancerrxgene/releases/release-8.2/ANOVA_results_GDSC1_20Feb20.xlsx and https://ftp.sanger.ac.uk/project/cancerrxgene/releases/release-8.2/ANOVA_results_GDSC2_20Feb20.xlsx. Both GDSC1 and GDSC2 datasets were downloaded, merged and reshaped, giving as a result a matrix containing the IC50, defined as the drug concentration that reduces cell viability by 50%, values for 565 drugs across 988 cell lines. Additionally, a file containing the maximum concentration of each drug was created. Only cell lines for which there was data from both CRISPR and drug sensitivity screens were considered, thus resulting in a subsample of 542 cell lines. Moreover, only compounds that displayed an IC50 lower than half on the maximum screened in at least three cell lines were considered. The cell line annotation file we used for ID conversion can be found at the following URL: https://cog.sanger.ac.uk/cmp/download/model_list_20230608.csv.

#### Linear mixed model (LMM)

According to the protocol, associations between drug response and gene fitness scores were performed using a mixed effect linear model (*limix* package in python, version 3.0.1), considering the following covariates: (i) binary variables indicating the institute of origin, (ii) principal component 1 of the drug response dataset and (iii) growing conditions represented by binary variables.

The random effects are represented by the gene fitness similarity matrix of the samples.

The final model fitted is the following:

$$d={\beta }_{0}M+{\beta }_{1}e+\mu X+\varepsilon$$

d represent the vector of drug response IC50 values, M represents the matrix of covariates stated above, β_0_ represent the vector of effect sizes, *e* represents the vector of fold changes, β_1_ represents the effect size, X represents the similarity matrix based on the CRISPR-cas9 gene fitness measurements, μ represents the random effects, ε represents the general noise term.

The significance of each association was computed by performing likelihood-ratio between the alternative model, which leverage CRISPR gene sensitivity measurements to explain the drug response, and the null model, which exclude those data and its parameters to explain the drug sensitivity measurements.

All pairwise associations between 426 compounds and 17,486 genes were tested, resulting in a total of 7,449,036 associations. P-value adjustments for multiple comparisons were performed by using the false discovery rate (FDR) procedure.

Considering an FDR-adjusted *P*-value < 0.1, 771 significant associations between drug response and gene fitness profiles were identified and used for further analysis.

### Method to identify drug resistance

In order to quantify the discordance of each cell line from the “canonical” response, the orthogonal distance from each point to the regression line, proper of each association, was computed across the 771 significant associations, resulting in a 771 × 542 distance matrix.

Given a point with coordinates (X_0_, Y_0_) and the equation of the regression line in standard notation: Ax + By + C = 0, the distance was computed as

$$d = \frac{\left|A{x}_{0}+B{y}_{0}+C\right|}{\sqrt{{A}^{2}+{B}^{2}}}$$

In the distance matrix, each column is a pair of gene-drugs while each row is a cell line, and each entry is the orthogonal distance of the cell line from the regression line of a particular association.

The next step is to identify cell lines which have a positive distance across many associations, thus showing a decreased sensitivity to drug response with respect to gene sensitivity. The association in the distance matrix includes a wide range of drug classes. Therefore, in order to identify mechanisms which confer resistance to those drugs, it is reasonable to group associations into clusters of drugs which act on the same pathway.

The information needed to perform association clustering according to the drug’s specific pathways of action was obtained from the *drugsheet* worksheet in the “Dataset-EV2” supplementary file from Gonçalves et al. Using the additional information for common drugs, we have selected 15 drug’s pathway and used each one of them to group the distance matrix column. In those restricted distance matrices, the median distance value of each cell line across selected associations was calculated and sorted taking into account the sign of the distance. Top 10% of cell lines with positive distances were selected for differential gene expression analysis with respect to the 10% cell lines with distance closest to 0.

### Differential expression and Functional Enrichment Analysis

Gene expression data was downloaded from Cell Model Passport^28^. Version 20210329 of the Sanger and Broad merged RNA-seq data was used (https://cog.sanger.ac.uk/cmp/download/rnaseq_merged_Sanger_Broad_20210329.zip).

In order to perform the differential gene expression analysis (DEG) we used the Bioconductor package *Deseq2*^29^ to process the unnormalized version “read count” of the downloaded folder, following the *Deseq2* documentation recommendations. DEG analysis was performed for each cluster and then, genes with adjusted p-value < 0.01 and with abs(log_2_(FoldChange)) > 3 were selected for enrichment analysis. Enrichment analysis was performed using R package *gprofiler2*^30,31^ available from CRAN or conda-forge.

The analysis was conducted by considering the organism of origin and across all available data sources: Gene Ontology, KEGG, Reactome, WikiPathways, TRANSFAC, miRTarBase, Human Protein Atlas, CORUM, Human phenotype ontology.

### Identification of cell lines involved in the resistance to drug treatment

#### Data description

The expression data used for the first part of our analysis was downloaded from two different platforms: Cell Model Passport^28^ and DepMap^15^. We used the data downloaded from the two platforms to cross check the results. Both datasets were first filtered to extract only the 542 cell lines of interest (obtained as described in the previous section).

The data downloaded from DepMap were already normalized using a log_2_(TPM + 1) normalization; while from Cell Model Passport a folder containing three versions of the data (raw data, TPM normalization and FPKM normalization) was downloaded. The TPM normalization dataset was converted in log_2_(TPM + 1), the raw data were used to perform the log_2_(DESeq + 1) normalization, while the FPKM normalization was not used.

Two types of normalization were used (TPM and DESeq) because we could not anticipate which method was going to provide better results for our analysis. The summary of the datasets used is in Table 3.

**Table 3:** Summary of the datasets used.

	Downloaded from	Type of normalization	Dimension (cell lines × genes)
Dataset 1	Cell model passport	log_2_(TPM + 1)	541 × 37,262
Dataset 2	Cell model passport	log_2_(DESeq + 1)	541 × 37,262
Dataset 3	DepMap	log_2_(TPM + 1)	474 × 19,177

#### List of genes causing MDR

Looking into the literature a list of genes involved in Multidrug resistance (MDR) was created (Table 4). The aim was to create a list of genes in which the genes causing drug resistance are not related with the mechanism of action of the drug. For this reason, the main focus was on genes related with Drug Efflux^4,7,8,32^.

**Table 4:** List of Multidrug Resistant genes.

	MDR genes
Drug efflux	ABCB1
	ABCC1
	ABCC10
	ABCG2
	ABCC3
	ABCB2
	ABCC2
	ABCB4
	ABCB11
	ABCC5
	MVP

#### UMAP

To visualize the data downloaded from Cell Model Passport and from DepMap we used the Uniform Manifold Approximation and Projection (UMAP) technique. This analysis was done using the R package *umap*^33^ and the resulting graphs are reported in Supplementary Figure 1.

### Identification of drug resistant cell lines

The aim of this step was to identify drug resistant cancer cell lines. This could be done by identifying the cell lines in which one or more genes recognized as MDR are highly expressed. To do this the biclustering approach was investigated. In particular, this analysis was done using the ISA method and the corresponding R package available (*isa2*^34^).

For this analysis the input for the function *isa* was a subset of the expression dataset considering only the genes involved with Drug Efflux. Moreover, it was used with the option *direction* = *“up”*. In this way we obtained clusters in which the expression of the gene was higher with respect to the mean. This analysis was done three times using the three datasets described in the Data description.

#### Intersection with the list of drug resistant cell lines previously identified

The goal of this step was to check that there is a concordance between the drug resistant cell lines we identified, and the drug resistant cell lines identified in the previous work as described in the previous section. To do this the Chi-square test was performed using the R function *chisq.test* with a significance level of 5%.

The contingency table used to compute the Chi-square test was created using as variables the drug resistant cell lines identified in the previous work and the drug resistance cell lines identified with the biclustering approach.

The cell lines we identified as drug resistance were the cell lines present in the ISA clusters. The Chi-square test was done using different combinations of the lists of resistant cell lines:

1. Union of all cell lines in bicluster versus list of resistant cell lines previously identified.
2. Union of all cell lines in bicluster versus list of resistant cell lines previously identified and grouped by MoA.

The cell lines identified as drug resistant from the previous work were arbitrarily chosen according to the distance to the regression line. In this case several numbers of drug resistant cell lines (top N cell line with a positive distance from the regression line) were used.

### Investigating the mechanisms that induce resistance to drug treatment and CRISPR-Cas9 in cancer cell lines

#### Data collection and preparation

To build the GRN the cancer cell lines were divided into resistant and non-resistant cell lines according to their distance from the regression line: top 100 cell lines with a positive distance from the regression line are the resistant cell lines, all the others are the non-resistant cell lines for the analysis of the resistance to chemotherapeutics while bottom 100 cell lines with a negative distance from the regression line are the resistant cell lines, all the others are the non-resistance cell lines for the analysis of the resistance to CRISPR-Cas9. The cell lines were also divided according to the tissue of origin. For first analysis only cell lines from the Lung and the Large Intestine were selected.

To build the GRNs, expression data and SNP information for each single cell line were needed. This information was taken from the GEO repository^35^ (accession number GSE36139). All the downloaded files were in CEL format. To convert data in the CEL files, several R libraries were used depending on data type. For the expression data the library used to convert the CEL file was *Affy*^36^ and the annotation file downloaded from BrainArray^37^ was *hgu133plus2hsentrezgcdf*. The position of the genes was extracted using the library *biomaRt*^38^. For the SNP data the library used to convert the CEL file was *crlmm*^39^ while the position of the SNPs was extracted using the file *GenomeWideSNP_6.na35.annot.csv*.

The first step performed to build GRN was the identification of the differentially expressed genes between resistant cell lines and non-resistant cell lines. The selected cell lines were ordered according to their median distance from the regression lines (Supplementary Table 3). The top 20% cell lines with a positive distance from the regression line and the 20% cell lines closest to the regression line were used to investigate the drug resistance, while the bottom 20% cell lines with a negative distance from the regression line and the 20% cell lines closest to the regression line were used to investigate the CRISPR-Cas9 resistance. In the latter analysis the last cell line SIDM00704 (Supplementary Table 2) was not considered since its median value was found to be too close to the value of the bottom cell lines. Direct comparison of the extreme subgroups was performed to highlight the differences between resistant and non-resistant cell lines. For this analysis, the raw expression data taken from the Cell Model Passport was utilized. The DEGs were identified using the R library *limma*^40^ and setting the threshold of significance at *p*-value < 0.05. For the analysis of chemotherapy resistance, we obtain 1615 genes differentially expressed in Lung, and 1250 genes in Large Intestine, while for the analysis of the CRISPR-Cas9 resistance we obtain 1329 genes differentially expressed in Lung.

Next step was to run the R library *MatrixEQTL*^41^ to identify the cis eQTL. To run *MatrixEQTL* all cell lines (primary tumor + metastasis) of the selected tissue are used. In this way the number of possible cis eQTL obtained is very large (on the order of 10^5^). The function *Matrix_eQTL_main* takes in input the expression data, the position of the genes, the SNP matrix, the position of the SNPs, and the covariance matrix then returns as output the information about the cis eQTLs. The model used to run the function was the linear regression model while the parameters needed were setted as follows: pvOutputThreshold.cis = 0.99, pvOutputThreshold = 0, cisDist = 1e6.

The eQTLs obtained must go through some filtering steps: (1) filter for FDR, (2) filter for minor allele frequency (MAF > 0.01), (3) identify linearly independent SNPs. In this way the uninformative SNPs were removed.

For the first filtering step different FDR values were used: for the analysis of the drug resistance we used an FDR < 0.05 in Lung and and FDR < 0.2 in Large Intestine while for the analysis of the CRISPR-Cas9 resistance we used an FDR < 0.1. Different values of FDR were empirically chosen to obtain a sufficiently large GRN. After these filtering steps, we ended with 18 genes and 22 SNPs for the study of drug resistance in the Lung, 11 genes and 11 SNPs for the study of drug resistance in the Intestine and 25 genes and 31 SNPs for the study of CRISPR-Cas9 resistance in the Lung. The obtained genes and SNPs were then used to build the GRN.

All the steps to build the GRN are summarized in Supplementary Fig. 5, while in Supplementary Table 8 the number of cell lines used for each step of the analysis are summarized.

#### Gene regulatory network construction

The gene regulatory network was computed using the R package *fssemR*^14^. For the construction of the gene regulatory network are used only primary tumor cell lines (not metastasis) of the selected tissue. In this way we are deleting all possible mutations that the cells can acquire to become metastatic.

The first step was the initialisation of the data with the Ridge regression using the functions *cv.multiRegression* to compute the gamma and *multiRegression* to compute the fit0. The second step was the running of the FSSEM algorithm itself with the function *opt.multiFSSEMiPALM2*.

The graphical visualization of the differential GRN between the two conditions (resistant cancer cell lines and non-resistant cancer cell lines) was done using the R package *network*^42^.

#### Gene regulatory network study

The study of the GRN obtained was done in two parts. First, the genes that appear in the GRN and connected in a subnetwork were studied singularly by searching in literature for each of them a known connection with drug resistance in cancer and with CRISPR-Cas9.

Second, the edges in the GRN were studied by uploading the input and the output gene in STRING^43^ and identifying the edges between the two genes in the GRN. When the direct edge was not present, the intermediate genes needed to connect the two genes in the GRN obtained were searched. For this second part, there are some genes, such as long noncoding genes that are not found on STRING. To study the connections between this gene and another, some research in literature about a possible relationship between the analysed genes was done.

#### Overlap between list of DEG and list of genes divided for MoA

For each MoA identified in the first part of this work we counted how many genes were also present in the list of DEG obtained as described in the *Data Collection and Preparation* section. Next, we checked if the overlap was significant using a Hypergeometric test with no multiple testing correction. This was performed using the R function *phyper*.

### Supplementary Information

Supplementary Information.

## Data Availability

The datasets employed in our study are available from the following repositories: Cell Model Passports and DepMap for the RNAseq data and for the CRISPR knockout data, Genomics of Drug Sensitivity in Cancer for the drug sensitivity data (GDSC1 and GDSC2 datasets), and Gene Expression Omnibus (GEO) for dataset GSE36139 comprising SNP and Expression data from the Cancer Cell Line Encyclopedia (CCLE). The URLs for the specific versions of the data files we used for our analysis are provided in the Methods section.
