# Supplementary material for: Investigating the mechanisms underlying resistance to chemotherapy and to CRISPR-Cas9 in cancer cell lines
Source: Sci Rep. 2024 Mar 5;14:5402. doi: 10.1038/s41598-024-55138-x (PMC10915165; doi:10.1038/s41598-024-55138-x)
Supplement: Supplementary file 1 — Supplementary Information. [file 41598_2024_55138_MOESM1_ESM.pdf]

SUPPLEMENTARY MATERIALS

Investigating the Mechanisms Underlying Resistance to Chemoterapy and to CRISPR-Cas9 in Cancer Cell Lines

Francesca Tomasi[1], Matteo Pozzi[1,2], and Mario Lauria[3,4*]

1 CIBIO Department, University of Trento, Povo, Italy
2 Fondazione Bruno Kessler, Povo, Italy
3 Department of Mathematics, University of Trento, Povo, Italy
4 Fondazione The Microsoft Research - University of Trento Centre for Computational and Systems Biology, Rovereto, Italy

*To whom correspondence should be addressed: mario.lauria@unitn.it.

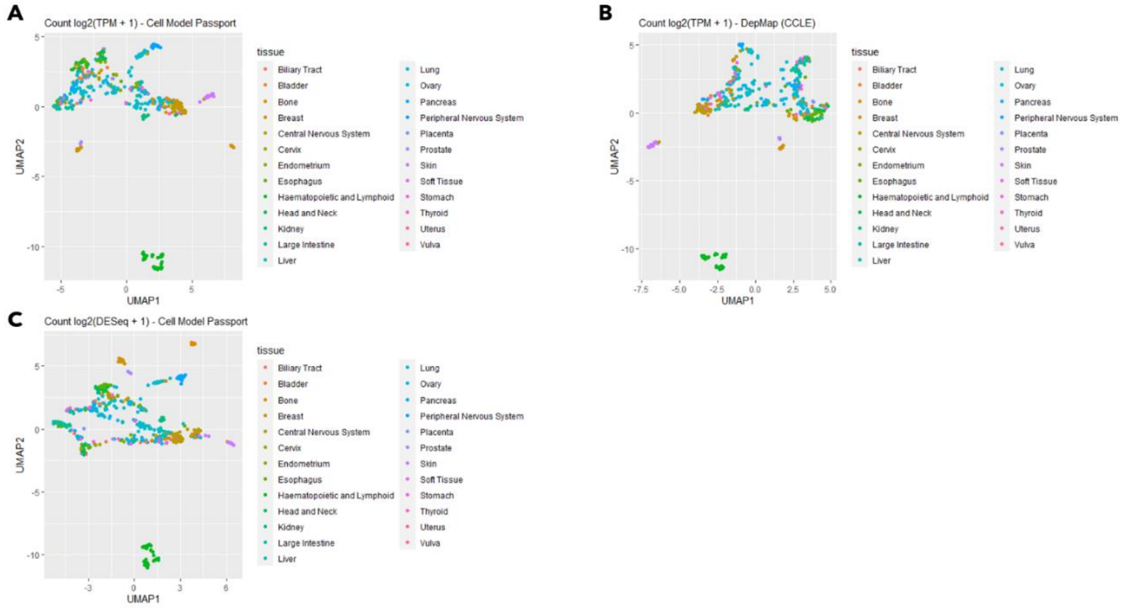

Supplementary Figure 1: UMAP representation considering all genes in the dataset. **A.** UMAP representation using the dataset downloaded from Cell Model Passport and normalized with log_2(TPM + 1). **B.** UMAP representation using the dataset downloaded from DepMap and normalized with log_2(TPM + 1). **C.** UMAP representation using the dataset downloaded from Cell Model Passport and normalized with log_2(DESeq + 1).

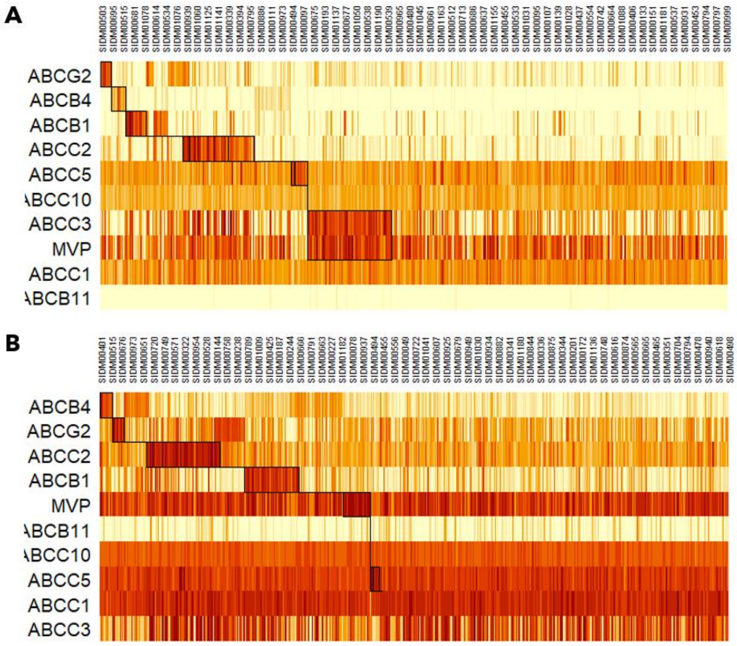

Supplementary Figure 2: Results of biclustering performed using the subset of MDR genes and the ISA method. **A.** Dataset downloaded from DepMap with normalization $\log_2$(TPM + 1). **B.** Dataset downloaded from Cell Model Passport with normalization $\log_2$(DESeq + 1).

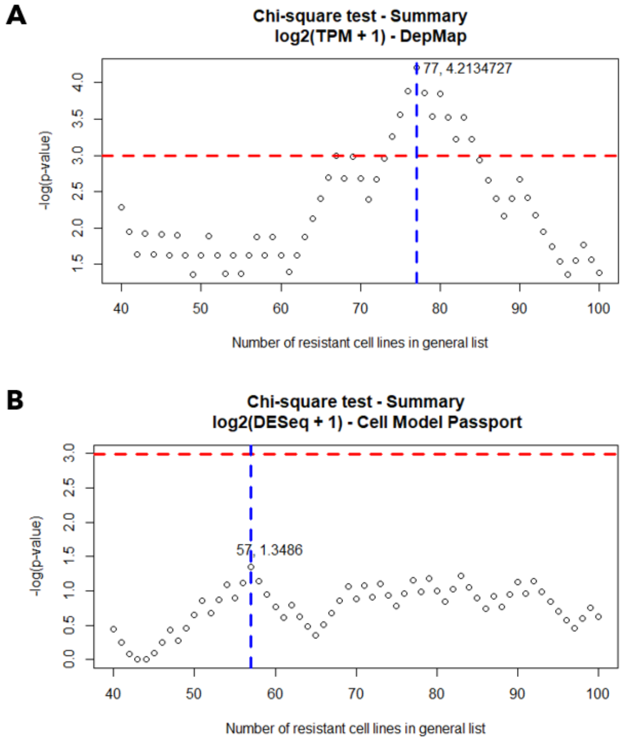

Supplementary Figure 3: Chi-square test results. The red line corresponds to p-val = 0.05. The blue line corresponds to the maximum value of the -log(p-value). The number next to the blue line is the number representing how many top N cell line are needed to have the maximum -log(p-value). **A.** Dataset downloaded from DepMap with normalization log_2(TPM + 1). **B.** Dataset downloaded from Cell Model Passport with normalization log_2(DESeq + 1).

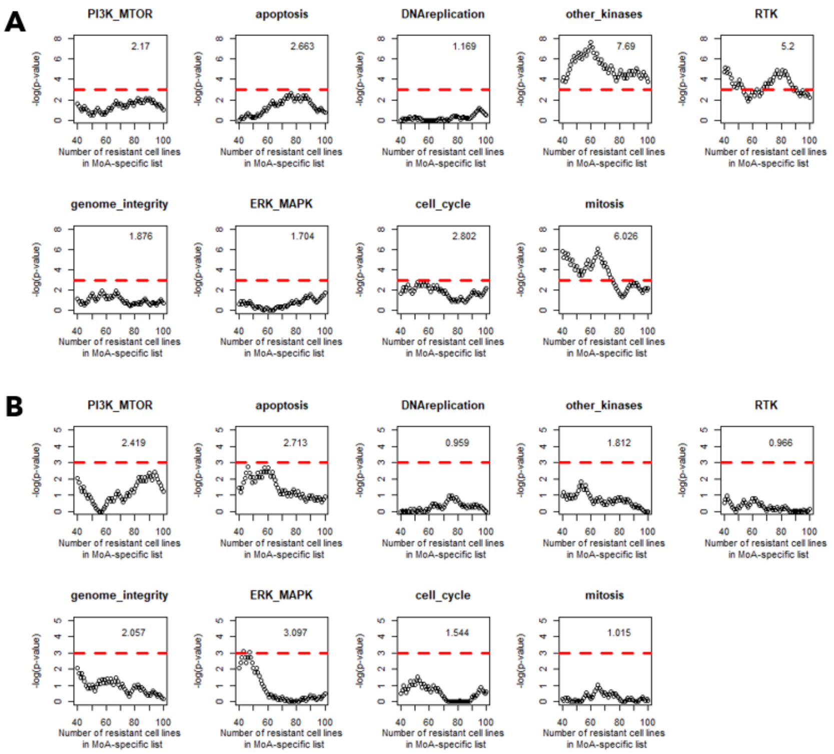

Supplementary Figure 4: Chi square test performed on the resistant cell lines identified for each mechanism of action and the sum of all resistant cell lines identified with the ISA biclustering method. The red line corresponds to p-val = 0.05. The numerical value inside each plot is the score of the highest -log(p-value). **A.** Dataset downloaded from DepMap with normalization $\log_2$(TPM + 1). **B.** Dataset downloaded from Cell Model Passport with normalization $\log_2$(DESeq + 1).

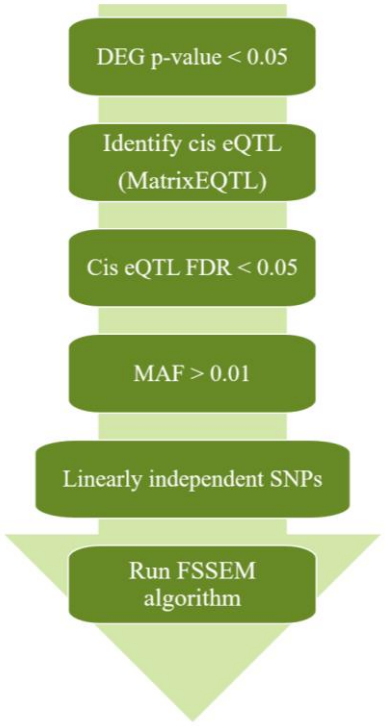

Supplementary Figure 5: Steps to build GRNs.

Dataset from	Normalization	Method	Nr of clusters	Dimension of biggest cluster	Dimension of smallest cluster
DepMap (CCLE)	log_2(TPM +1)	ISA	17	2x73	1x3
Cell Model Passport	log_2(TPM +1)	ISA	20	2x91	1x2
	log_2(DESeq +1)	ISA	25	2x48	1x4

Supplementary Table 1: Summary of Biclustering results using ISA method. In the table is present the corresponding number of clusters identified and the dimension of the biggest and smallest cluster for each dataset.

tissue	# resistant cell lines	# NON resistant cell lines
Biliary Tract	1	1
Bladder	2	11
Bone	1	18
Breast	5	29
Central Nervous System	10	34
Cervix	2	5
Endometrium	4	6
Esophagus	3	27
Haematopoietic and Lymphoid	2	48
Head and Neck	2	34
Kidney	2	10
Large Intestine	12	24
Liver	1	13
**Lung**	**28**	**65**
Ovary	5	27
Pancreas	11	16
Peripheral Nervous System	3	19
Placenta	0	2
Prostate	1	3
Skin	2	21
Soft Tissue	0	6
Stomach	3	17
Thyroid	0	4
Uterus	0	1
Vulva	0	1

Supplementary Table 2: List of tissues with the corresponding number of cell lines which are resistant and non-resistant to chemotherapy.

model_id	median
SIDM00926	0.812146188338355
SIDM00755	0.723848040735098
SIDM00119	0.54004171647714
SIDM00523	0.498324367453951
SIDM01163	0.442167012437328
SIDM01119	0.419488726962372
SIDM00117	0.34426055145408
SIDM00924	0.342254802780432
SIDM01124	0.331826183163243
SIDM00547	0.322262920830735
SIDM00116	0.302222555853682
SIDM01156	0.301859556121529
SIDM00300	0.283477387207412
SIDM01072	0.248804136096784
SIDM00921	0.238442035058102
SIDM01067	0.236066674877789
SIDM01134	0.221744027643328
SIDM00222	0.13119894464411
SIDM00133	0.12751357436447
SIDM01070	0.125240192485326
SIDM01130	0.123262589561214
SIDM00548	0.122666226770995
SIDM00722	0.114368261977176

model_id	median
SIDM00341	0.110837709603098
SIDM00486	0.0984839500173574
SIDM00138	0.0930914191396067
SIDM00137	0.0858535329679817
SIDM00354	0.0644154824496384
SIDM00716	0.0308789079033824
SIDM00294	-0.00373941174222974
SIDM01078	-0.0289935105963041
SIDM00748	-0.0682371624062657
SIDM00704	-0.10317216720943
SIDM00718	-0.107608356936935
SIDM00237	-0.108646452856526
SIDM00810	-0.122387814059769
SIDM00759	-0.133880730379185
SIDM00939	-0.146260379286052
SIDM00344	-0.194372180679642
SIDM00740	-0.215686862535926
SIDM00509	-0.235577572000193
SIDM00339	-0.252468927668985
SIDM00598	-0.276231973165668
SIDM00903	-0.298816510241508
SIDM01044	-0.367270438402616
SIDM01069	-0.371844490123228

Supplementary Table 3: List of lung – primary tumor cell lines ordered according to their median distance from the regression line. In blue are highlighted the top 20% LUNG – primary tumor cell lines identified as chemoresistant, in green are highlighted the 20% LUNG – primary tumor cell lines around the regression line and in grey are highlighted the bottom 20% LUNG – primary tumor cell lines identified as resistant to Crispr CAS9 mechanism.

Entrez ID	Gene Symbol	Chromosome	Description	Resistance
127933	UHMK1	Chr 1	UHMK1 is Serine/threonine kinase involved in the phosphorylation of proteins and the processing of RNA.	It is known that UHMK1 expression was positively related to the oxaliplatin resistance of colorectal cancer (CRC) cells. UHMK1 interacts with STAT3 influencing IL6/STAT3 signaling.
138046	RALYL	Chr 8	RALYL enables identical protein binding activity and is located in nucleoplasm.	RALYL could promote HCC tumorigenicity, self-renewal, chemoresistance, and metastasis. The expression of RALYL increase resistance to CDDP and 5-Fu in cancer cell lines.
153218	SPINK13	Chr 5	SPINK13 is predicted to enable serine-type endopeptidase inhibitor activity and to be involved in negative regulation of acrosome reaction.	SPINK13 is not known to play a role in drug resistance in cancer cells.<br>SPINK1 overexpression increases the aggressiveness of cancer cells and confers chemo-resistance to several drugs in pancreatic cancer. SPINK1 acts activating EGFR independent and dependent pathways.
400680	LINC00664	Chr 19	LINC00664 is an RNA gene affiliated with the lncRNA class.	Long noncoding RNAs (lncRNAs) can mediate carcinogenesis and drug resistance/sensitivity in cancer cells.<br>LINC00511 is up-expressed in many cancers and is related to several features of cancer among which drug resistance.<br>LINC01977 promotes breast cancer (BC) development and chemoresistance to doxorubicin.<br>LINC00173 is upregulated in chemoresistant small cell lung cancer (SCLC) cell lines.
4259	MGST3	Chr 1	This gene encodes an enzyme which catalyzes the conjugation of leukotriene A4 and reduced glutathione to produce leukotriene C4.	The overexpression of MGST3 was found in cisplatin resistant lung adenocarcinoma cells and spatial differences in MGST3 expression could be involved in chemoresistance.
64978	MRPL38	Chr 17	MRPL38 is a mammalian mitochondrial ribosomal protein encoded by nuclear genes which helps in protein synthesis within the mitochondrion.<br>MRPL38 expression is linked with invasion and metastasis.	MRPL38 is not known to play a role in drug resistance in cancer cells.<br>MRPS29 expression increase resistance to chemotherapy by inhibiting apoptosis and promoting cell migration.<br>MRPL12 as a downstream gene of c-Myc participates in the cetuximab resistance in RAS wild-type colorectal cancer cells.

Entrez ID	Gene Symbol	Chromosome	Description	Resistance
790952	ESRG	Chr 3	ESRG is an RNA gene associated with the lncRNA class. Diseases associated with ESRG include Embryonal Carcinoma and Germinoma.<br>ESRG is dispensable for the maintenance and recapturing of human pluripotency.	ESRG is not known to play a role in drug resistance in cancer cells.<br>ESRG is expressed in embryonic carcinoma cell. Therefore, part of the cancer cell lines are cancer stem cell (CSCs). CSCs have stemness and phenotypic plasticity properties that are responsible for resistance to chemotherapy, radiotherapy, metastasis, cancer development, and tumor relapse.
8239	USP9X	Chr X	USP9X is a member of the peptidase C19 family and encodes a protein that is similar to ubiquitin-specific proteases.	USP9X regulates BRCA1 which mediated HR repair and contributes to the development of resistance in cancer cells to DNA-damaging agents.
90187	EMILIN3	Chr 20	EMILIN3 enables identical protein binding activity. It is part of collagen-containing extracellular matrix.	EMILIN3 is not known to play a role in drug resistance in cancer cells.<br>MMRN1 (member of the EMILIN/multimerin family) downregulation is associated with chemoresistance in rectal cancer. MMRN1 was identified with other 16 genes significantly correlated to leukemia stemness and chemoresistance.

Supplementary Table 4: Description of the nodes of the GRN (cis eQTL FDR < 0.05, MAF > 0.01, and selection of linearly independent SNPs) describing mechanisms of drug resistance in cancer cell lines.

start	end	Chr	**Interaction mediated by**	**Notes**
EMILIN3	RALYL	RALYL is on Chr 8<br>EMILIN3 is on Chr 20	2 intermediate genes: EMILIN3, ZNRD1, BARX2, RALYL	RALYL enables **identical protein binding activity**. Located in nucleoplasm.<br>EMILIN3 enables **identical protein binding activity**. Part of collagen-containing extracellular matrix.
SPINK13	MGST3	MGST3 is on Chr 1<br>SPINK13 is on Chr 5	3 intermediate genes: SPINK13, SPINK9, SOD3, PTGES, MGST3	/
UHMK1	LINC00664	LINC00664 is on Chr 19<br>UHMK1 is on Chr 1	LINC00664 not found on STRING	UHMK1 and MGST3 are on the same Chr.<br>Addionally UHMK1 and MGST3 have the same *Top Transcription factor binding sites by QIAGEN*: Pbx1a.
MGST3	LINC00664	LINC00664 is on Chr 19<br>MGST3 is on Chr 1	LINC00664 not found on STRING	
ESRG	MRPL38	MRPL38 is on Chr 17<br>ESRG is on Chr 3	ESRG not found on STRING	MRPL38 and USP9X have the same *Top Transcription factor binding sites by QIAGEN in the gene promoter: Egr-4.*
ESRG	USP9X	USP9X is on Chr X<br>ESRG is on Chr 3	ESRG not found on STRING	
LINC00664	ESRG	ESRG is on Chr 3<br>LINC00664 is on Chr 19	ESRG and LINC00664 not found on STRING	These are two non-coding RNA.<br>Interact with the same miRNA: hsa-miR-1224-3p.

Supplementary Table 5: Connections present in the GRN which underlies the mechanisms that cause drug resistance.

Entrez ID	Gene Symbol	Chromosome	Description	CRISPR-Cas9
10005	ACOT8	Chr 20	ACOT8 is a members of the acyl-CoA thioesterase (ACOT) superfamily. It is involved in the oxidation of fatty acid, and catalyzes the hydrolysis of acyl-CoAs into free fatty acids and coenzyme A (CoASH), regulating their respective intracellular levels.	Experiments with CRISPR/Cas9 knockout cell lines for ACOT8 will be conducted in future.
10950	BTG3	Chr 21	BTG3 codes for a protein member of the BTG/Tob family. It is a downstream target of p53.	BTG3 knocked out in mice. The result is that BTG3 knockout mice appeared normal and were not sensitive to tumor formation.
114327	EFHC1	Chr 6	EFHC1 codes for an EF-hand-containing calcium binding protein, and it plays a role in calcium homeostasis.	Experiments in which EFHC1 is engineered using CRISPR-Cas9 are not found in literature.
201501	ZBTB7C	Chr 18	ZBTB7C facilitates DNA-binding transcription factor activity, RNA polymerase II-specific and RNA polymerase II cis-regulatory region sequence-specific DNA binding activity. It is involved in negative regulation of cell population proliferation.	Experiments in which ZBTB7C is engineered using CRISPR-Cas9 are not found in literature.
2135	EXTL2	Chr 1	EXTL2 is a member of the EXT family. It can act as alpha-1,4-N-acetylgalactosaminyltransferase and as 4-alpha-N-acetylglucosaminyltransferase. EXTL2 can be involved in heparan sulfate (HS) elongation.	A knockout of EXTL2 is performed to show that this increase HS levels in cells
26099	SZRD1	Chr 1	SZRD1 is a protein coding gene which plays an important role in cell cycle. The overexpression of SZRD1 produces cell cycle arrest in G2 by upregulating P21.	Experiments in which SZRD1 is engineered using CRISPR-Cas9 are not found in literature.
323	APBB2	Chr 4	APBB2 codes for a protein which can bind to the β-amyloid precursor protein (βAPP), therefore can mediate APP processing. APBB2 include two phosphotyrosine binding (PTB) domains, which are supposed to have a role in signal transduction.	Experiments in which APBB2 is engineered using CRISPR-Cas9 are not found in literature.
3910	LAMA4	Chr 6	LAMA4 is a protein coding gene that belongs to the laminins. These proteins are involved in several biological processes as cell adhesion, differentiation, migration, signaling, neurite outgrowth and metastasis.	CRISPR-Cas9 is used to eliminate laminin a4 and b1 chains.

Entrez ID	Gene Symbol	Chromosome	Description	CRISPR-Cas9
51176	LEF1	Chr 4	LEF1 is a transcription factor involved in the Wnt signaling pathway. It can operate in hair cell differentiation and follicle morphogenesis. The protein coded by LEF1 can bind a functionally important site in the T-cell receptor-alpha enhancer, therefore this confers maximal enhancer activity.	Several article in which CRISPR-Cas9 is used to knockout LEF1 are present in literature. CRISPR-Cas9 technology is used to insert LEF1 into an adenovirus-associated virus (AAVS1) integration locus on chromosome 19 into human umbilical cord-derived mesenchymal stem cells.
53833	IL20RB	Chr 3	IL20RB together with IL20RA form a heterodimeric receptor for interleukin-20.	Knock down of IL20RB is done in control ID8 cells and IL20RA-reconsitituted ID8 cells. They found out that the silencing of IL20RB prevents the activation of the STAT3/NLR signaling in IL20RA-reconstituted ID8 cells.
65990	ANTKMT	Chr 16	ANTKMT (FAM173A) facilitates protein-lysine N-methyltransferase activity and is needed in peptidyl-lysine trimethylation.	FAM173A knockout invalidate lysine methylation of a single mitochondrial protein in human cells and allows to identify Lys-52 as targets of FAM173A in ANT2 and ANT3.
80273	GRPEL1	Chr 4	GRPEL1 is a component of the PAM complex thus is implicated in protein import into mitochondrial matrix. This gene facilitates identical protein binding activity and unfolded protein binding activity.	CRISPR/Cas9-based editing is tested on GRPEL genes. They were capable to produce independent clones lacking GRPEL2, but no GRPEL1 knockout clones were obtained.
862	RUNX1T1	Chr 8	RUNX1T1 is a member of the myeloid translocation gene (MTG) family. The genes of this family interact with DNA-bound transcription factors and recruit a range of corepressors to facilitate transcriptional repression. RUNX1T1 itself is a transcription factor and has a role as a transcriptional modulator.	Knockout of RUNX1T1 using CRISPR-Cas9 technology shows that this gene increase the aggressiveness of neuroblastoma cells.
91464	ISX	Chr 22	ISX is a transcription factor that regulates gene expression in intestine and a homeobox genes which encode DNA-binding proteins. Additionally, this gene is a proto-oncogene which regulates cell proliferation, and drives HCC formation.	Experiments in which ISX is engineered using CRISPR-Cas9 are not found in literature.

Supplementary Table 6: Description of the nodes of the GRN (cis eQTL FDR < 0.1, MAF > 0.01, and selection of linearly independent SNPs) describing mechanisms of CRISPR-Cas9 resistance in cancer cell lines.

end	start	Interaction mediated by	Type of connection	Notes
LAMA4	ACOT8	1 intermediate gene: ACOT4	LAMA4 - ACOT4: putative homologs are mentioned together in other organisms & putative homologs are co-expressed in other organisms.<br>ACOT4- ACOT8: are co-mentioned in pubmed abstract & have an association in Curated Databases & putative homologs are co-expressed in other organisms.	
LAMA4	BTG3	1 intermediate gene: LEF1	LAMA4 - LEF1: co-mentioned in pubmed abstract.<br>LEF1 - BTG3: co-mentioned in pubmed abstract & putative homologs were found interacting in other organisms.	
RUNX1T1	BTG3	1 intermediate gene: TCF12	RUNX1T1 - TCF12: are co-mentioned in pubmed abstract & are present in Experimental/Biochemical Data & putative homologs are co-expressed in other organisms.<br>TCF12 - BTG3: are co-mentioned in pubmed abstract.	3
ISX	BTG3	1 intermediate gene: LEF1	ISX - LEF1: are co-mentioned in pubmed abstract & putative homologs were found interacting in other organisms.<br>LEF1 - BTG3: co-mentioned in pubmed abstract & putative homologs were found interacting in other organisms.	
BTG3	ZBTB7C	2 interemdiate gene: GNG13, APLP1	BTG3 - GNG13: are-mentioned in pubmed abstract.<br>GNG13 - APLP1: are co-expressed.<br>APLP1 - ZBTB7C: are co-mentioned in pubmed abstract.	
LEF1	ZBTB7C	1 intermediate gene: HDAC1	LEF1 - HDAC1: are co-mentioned in pubmed abstract & have an association in curated databases & are present in Experimental/ Biochemical Data.<br>HDAC1 - ZBTB7C: putative homologs are mentioned together in other organisms & putative homologs were found interacting in other organisms.	
EFHC1	EXTL2	2 intermediate gene: GABRD, EXTL3	EFHC1 - GABRD: are co-mentioned in pubmed abstract & putative homologs are co-expressed in other organisms.<br>GABRD - EXTL3: are co-mentioned in pubmed abstract & putative homologs are co-expressed in other organisms.<br>EXTL3 - EXTL2: are co-mentioned in pubmed abstract & have an association in curated databases & putative homologs were found interacting in other organisms & putative homologs are co-expressed in other organisms.	
BTG3	APBB2	2 intermediate genes: CHEK1, EGFR	BTG3 - CHEK1: are co-mentioned in pubmed abstract & are present in Experimental/ Biochemical Data & are co-expressed.<br>CHEK1 - EGFR: are co-mentioned in pubmed abstract.<br>EGFR - APBB2: are co-expressed & are present in Experimental/Biochemical Data & putative homologs are mentioned together in other organisms.	
IL20RB	APBB2	2 intermediate genes: IL10, APP	IL20RB - IL10: are co-mentioned in pubmed abstract & have an association in curated databases & putative homologs are co-expressed in other organisms.<br>IL10 - APP: are co-mentioned in pubmed abstract.<br>APP - APBB2: are co-mentioned in pubmed abstract are present in Experimental/ Biochemical Data & are co-expressed.	1

ISX	APBB2	2 intermediate genes: EGFR, RASD2	APBB2 - EGFR: are co-expressed & are present in Experimental/Biochemical Data & putative homologs are mentioned together in other organisms.<br>EGFR - RASD2: are co-mentioned in pubmed abstract & are co-expressed & putative homologs were found interacting in other organisms.<br>RASD2 – ISX: are co-mentioned in pubmed abstract & putative homologs were found interacting in other organisms.	Belong to the same GO pathway: regulation of transcription, DNA-dependent
IL20RB	LAMA4	2 intermediate genes: IL20RA, ITGB4	IL20RB - IL20RA: are co-mentioned in pubmed abstract & have an association in curated databases & are present in Experimental/Biochemical Data.<br>IL20RA - ITGB4: are co-mentioned in pubmed abstract & are co-expressed.<br>ITGB4 - LAMA4: are co-mentioned in pubmed abstract & have an association in curated databases & putative homologs were found interacting in other organisms & putative homologs are co-expressed in other organisms.	2
GRPEL1	LAMA4	1 intermediate gene: DNAJB1	GRPEL1 - DNAJB1: are co-mentioned in pubmed abstract & are co-expressed & are present in Experimental/Biochemical Data & homologous genes are neighbors in other genomes.<br>DNAJB1 - LAMA4: putative homologs are mentioned together in other organisms & putative homologs are co-expressed in other organisms.	
ACOT8	LEF1	2 intermediate geens: ACOT2, HNF4A	ACOT8 - ACOT2: are co-mentioned in pubmed abstract & & have an association in curated databases & putative homologs are co-expressed in other organisms.<br>ACOT2 - HNF4A: are co-mentioned in pubmed abstract & putative homologs are co-expressed in other organisms.<br>HNF4A - LEF1: are co-mentioned in pubmed abstract & putative homologs were found interacting in other organisms.	
APBB2	LEF1	1 intermediate gene: SMAD4	APBB2 - SMAD4: are co-mentioned in pubmed abstract & are present in Experimental/Biochemical Data.<br>SMAD4 - LEF1: are co-mentioned in pubmed abstract & have an association in curated databases & are present in Experimental/ Biochemical Data.	Belong to the same GO: negative regulation of apoptotic process
ACOT8	IL20RB	2 intermediate genes: ACOT2, IL24	ACOT8 - ACOT2: are co-mentioned in pubmed abstract & have an association in curated databases & putative homologs are co-expressed in other organisms.<br>ACOT2 - IL24: are co-mentioned in pubmed abstract.<br>IL24 - IL20RB: are co-mentioned in pubmed abstract & have an association in curated databases & are present in Experimental/ Biochemical Data & putative homologs are co-expressed in other organisms.	
EXTL2	IL20RB	2 intermediate genes: XBP1, IL24	EXTL2 - XBP1: have an association in curated databases.<br>XBP1 - IL24: are co-mentioned in pubmed abstract.<br>IL24 - IL20RB: are co-mentioned in pubmed abstract & have an association in curated databases & are present in Experimental/ Biochemical Data & putative homologs are co-expressed in other organisms.	

APBB2	IL20RB	2 intermediate genes: APP, IL10	APBB2 - APP: are co-mentioned in pubmed abstract are present in Experimental/ Biochemical Data & are co-expressed.<br>APP - IL10: are co-mentioned in pubmed abstract.<br>IL10 - IL20RB: are co-mentioned in pubmed abstract & have an association in curated databases & putative homologs are co-expressed in other organisms.	Inverse connection of 1
LAMA4	IL20RB	2 intermediate genes: ITGB4, IL20RA	LAMA4 - ITGB4: are co-mentioned in pubmed abstract & have an association in curated databases & putative homologs were found interacting in other organisms & putative homologs are co-expressed in other organisms.<br>ITGB4 - IL20RA: are co-mentioned in pubmed abstract & are co-expressed.<br>IL20RA - IL20RB: are co-mentioned in pubmed abstract & have an association in curated databases & are present in Experimental/Biochemical Data.	Inverse connection of 2
LEF1	IL20RB	2 intermediate genes: CTNNB1, IL24	LEF1 - CTNNB1: are co-mentioned in pubmed abstract & have an association in curated databases & are present in Experimental/ Biochemical Data & putative homologs are co-expressed in other organisms.<br>CTNNB1 - IL24: are co-mentioned in pubmed abstract.<br>IL24 - IL20RB: are co-mentioned in pubmed abstract & have an association in curated databases & are present in Experimental/ Biochemical Data & putative homologs are coexpressed in other organisms.	
SZRD1	ANTKMT	1 intermediate gene: METTL21A	SZRD1 - METTL21A: are co-mentioned in pubmed abstract.<br>METTL21A – ANTKMT: are co-mentioned in pubmed abstract.	
BTG3	RUNX1T1	1 intermediate gene: TCF12	BTG3 - TCF12: are co-mentioned in pubmed abstract.<br>TCF12 - RUNX1T1: are co-mentioned in pubmed abstract & are present in Experimental/Biochemical Data & putative homologs are co-expressed in other organisms.	Inverse connection of 3
ZBTB7C	RUNX1T1	1 intermediate gene: NCOR2	ZBTB7C - NCOR2: putative homologs are mentioned together in other organisms & putative homologs were found interacting in other organisms.<br>NCOR2 - RUNX1T1: are co-mentioned in pubmed abstract & are present in Experimental/Biochemical Data & putative homologs are co-expressed in other organisms.	
LAMA4	RUNX1T1	2 intermediate genes: ITGB3, RUNX1	LAMA4 – ITGB3: are co-mentioned in pubmed abstract & are co-expressed & have an association in Curated Databases & putative homologs were found interacting in other organisms.<br>ITGB3 - RUNX1: are co-mentioned in pubmed abstract & are co-expressed.<br>RUNX1- RUNX1T1: are co-mentioned in pubmed abstract & have an association in Curated Databases & are present in Experimental/Biochemical Data.	Belong to the same KEGG pathway: Pathways in cancer

Supplementary Table 7: Connection present in the GRN which underlies the mechanisms that causes CRISPR-Cas9 resistance. For some of these edges there are more than one possible combination of intermediate genes. In this table are presented the intermediate genes that have a more solid known connection with the genes of the network.

Cell type and analysis performed	Source	Resistant cell lines	Sensitive cell lines	Total cell lines
LUNG – primary tumor DRUG RESISTANCE	Cell Model Passport/DepMap	13	33	46
	GSE36139	8	28	36
LUNG – primary tumor + metastasis DRUG RESISTANCE	Cell Model Passport/DepMap	28	65	93
	GSE36139	21	58	79
LUNG – primary tumor CRISPR RESISTANCE	Cell Model Passport/DepMap	7	39	46
	GSE36139	7	29	36
LUNG – primary tumor + metastasis CRISPR RESISTANCE	Cell Model Passport/DepMap	13	80	93
	GSE36139	12	67	79
INTESTINE – primary tumor DRUG RESISTANCE	Cell Model Passport/DepMap	6	18	24
	GSE36139	5	16	21
INTESTINE – primary tumor + metastasis DRUG RESISTANCE	Cell Model Passport/DepMap	12	24	36
	GSE36139	10	21	31

Supplementary Table 8: Number of cell lines for each analysis.
